# Hypercholesterolemia and Myocardial function evaluated via Tissue Doppler Imaging

**DOI:** 10.1186/1476-7120-7-56

**Published:** 2009-11-27

**Authors:** Jack Rubinstein, Augusta Pelosi, Ameeth Vedre, Pavan Kotaru, George S Abela

**Affiliations:** 1 Department of Internal Medicine, Division of Cardiovascular Diseases, University of Cincinnati, Cincinnati, OH 45219, USA; 2 College of Veterinary Medicine, Small Animal Clinical Sciences, Michigan State University, East Lansing, MI 48824, USA; 3 Department of Medicine, Division of Cardiology, Michigan State University, East Lansing, MI 48824, USA; 4 Department of Medicine, Division of General Internal Medicine, Michigan State University, East Lansing, MI 48824, USA

## Abstract

**Objective:**

To establish a link between hypercholesterolemia and myocardial dysfunction.

**Background:**

Heart failure is a complex disease involving changes in systolic and diastolic function. Newer echocardiographic imaging modalities may be able to detect discreet changes in myocardial function associated with hypercholesterolemia. Therefore we sought to establish a link between hypercholesterolemia and myocardial dysfunction with tissue Doppler imaging (TDI).

**Methods:**

Twenty-seven rabbits were studied: 7 were fed normal chow (group 1) and 20 a high cholesterol diet (10 with ezetimibe, 1 mg/kg/day; group 2 and 10 without, group 3). Echocardiographic images were obtained under general anesthesia. Serum cholesterol levels were obtained at baseline, 3 and 6 months and myocardial cholesterol levels measured following euthanasia.

**Results:**

Doppler measurements, including E/A, E'/A' and S' were significantly lower in group 3 compared to both groups 1 and 2 but no significant differences were noted in chamber sizes or ejection fraction among the groups. Average serum cholesterol was higher in group 3 compared to groups 1 and 2 respectively (495 ± 305 mg/dl vs. 114 ± 95 mg/dl and 87 ± 37 mg/dl; p < 0.01). Myocardial cholesterol content was also higher in group 3 compared to group 2 (0.10 ± 0.04 vs. 0.06 mg/dl ± 0.02; p = 0.05). There was significant correlation between S', E'/A', E/E' and serum cholesterol (r^2 ^= 0.17 p = 0.04, r^2 ^= 0.37 p = 0.001 and r^2 ^= 0.24 p = 0.01).

**Conclusion:**

Cholesterol load in the serum and myocardium was significantly associated with decreased systolic and diastolic function by TDI. Moreover, lipid lowering was protective.

## Background

There are over five million cases of heart failure cases in the United States causing an estimated death rate of over a quarter of a million per year [1]. This complex condition encompasses both systolic and diastolic dysfunction with varying overlap between them [2]. Hypercholesterolemia is a well established risk factor for coronary artery disease [3] and is related to endothelial dysfunction at both the macro and microvascular level [4]. Furthermore, decreasing serum cholesterol levels with exercise, diet and medications has been associated in numerous studies with improved outcome [5]. But, the relationship of cholesterol and direct myocardial function has not yet been established. There have been several studies that have paradoxically demonstrated that hypercholesterolemia actually has a positive effect in patients with heart failure [6]. Furthermore, several studies that have attempted to improve outcomes with cholesterol lowering medications in patients with heart failure have shown either neutral or negative results [7]. Several theories have been proposed to explain these findings, but none have been accepted by the medical community nor thoroughly investigated [8]. In order to elucidate this we tested the hypothesis that cholesterol affects myocardial function. We sought to correlate serum and myocardial cholesterol levels with advanced echocardiographic parameters.

Echocardiography is a well-established clinical method used to evaluate patients with heart failure [9]. Recently, tissue Doppler imaging (TDI) has become more widely used to assess left ventricular (LV) systolic and diastolic function [10]. Parameters of diastolic function such as early diastolic velocities measured as E prime (E'), the E' to late diastolic filling (A') ratio (E'/A') and the transmitral to mitral annular early diastolic velocity ratio (E/E') [11] have all been shown to predict mortality and cardiovascular events [12]. The peak systolic velocity (S') is also a sensitive marker for mild LV systolic impairment, even in patients with a normal LV systolic function [13].

## Methods

### Hypercholesterolemic Rabbit Model

Twenty-seven adult male New Zealand White rabbits were studied, 7 were fed normal rabbit chow (group 1) and the remaining 20 were fed a high cholesterol diet as previous described [14,15] for 6 months. Briefly, rabbits were fed a high cholesterol diet (1%, Harlan-Sprague Dawley, Inc, Indianapolis, IN) alternating with normal chow on a monthly basis, 10 were treated with ezetimibe (1 mg/kg/day) (group 2) throughout the 6 months and the remaining were not (group 3). All studies were performed with the approval of Michigan State University Animal Care and Use Committee on Animal Investigations following NIH guidelines.

### Echocardiographic Images

After 6 months echocardiographic images were obtained after inducing general anesthesia with ketamine (15 - 25 mg/kg) and xylazine (3 - 5 mg/kg). Food was withheld overnight and the rabbits were placed in prone position without restraint.

All images were obtained with a 7 MHz probe (Vivid 7/Vingmed General Electric, Milwaukee, WI). Parasternal and apical views were obtained with the probe positioned at a cranial angle over a shaved area on the lower portion of the thoracic wall as previously reported [16,17]. The exact position of the transducer was adjusted as necessary to acquire standard images. Echocardiographic measurements by both 2D and M-mode were obtained in the parasternal long axis view. These included septal and posterior left ventricular (LV) wall thickness, LV cavity size (end-diastolic (LVEDD) and end-systolic (LVESD) dimensions), aortic root, and left atrial anteroposterior diameter. Fractional shortening was computed as follows: [(LVEDD-LVESD)/LVEDD] × 100 and as global systolic function was balanced the ejection fraction was calculated with the Teicholz formula [18].

Doppler imaging of the mitral valve was obtained from the apical four-chamber view. The following measurements were obtained at the mitral inflow velocity image: peak E and peak A wave velocities, E/A ratio, E wave acceleration and deceleration times, and isovolumetric relaxation time.

TDI was performed from an apical four-chamber view with very low contrast and a 5 mm tissue sampling volume at the mitral annulus from both septal and lateral walls. From the acquired images, the following diastolic function parameters were measured: E', A', E'/A' and E/E' wave ratio; the systolic parameter measured was S'.

All the measurements were obtained at the time of examination by the echosonographer and subsequently read by two separate physicians who were blinded to the groups, the average of the three measurements was used for subsequent analysis.

### Cholesterol analysis

#### Serum

Blood samples were drawn from ear veins with a 25-gauge needle and syringe at baseline and 3 months into the protocol. The samples were evaluated for cholesterol content using a cholesterol kit (Thermo Electron Corporation, Louisville, CO) according to the manufacturer's instruction.

#### Myocardial tissue

All rabbits were euthanized at six months (Euthonasol 1 cc IV) and the hearts removed. Blood was washed out of the cavity and a segment of myocardial tissue (10 × 3 mm) from the LV free wall was excised. Cholesterol levels were measured in the tissue segment using a cholesterol Kit (EnzyChrom Cholesterol Assay Kit (ECCH-100) BioAssay Systems, Hayward, CA).

### Statistical Analysis

GraphPad InStat 3.06 (GraphPad Software, San Diego, CA, USA) was used for statistical analysis. One way ANOVA was used to compare the E/A, E'/A', E/E', S', myocardial and serum cholesterol levels for all three groups. Pearson Correlation analysis was done between echocardiographic, TDI variables; and serum and myocardial cholesterol levels. A two tailed P value of < 0.05 was noted to have statistically significant value.

## Results

### Echocardiographic Findings

A full echocardiographic study was obtained in 24/27 rabbits (89%); TDI were not obtainable in three due to imaging difficulties involving adequate image resolution at the mitral annulus. The 2-D and M-mode measurements were similar and consistent without significant differences among all three groups and with the published data [17] (Table 1), and there were no significant differences noted between the independent measurements obtained by the echosonographer and the blinded readers.

**Table 1:** Comparison of published echocardiographic parameters in rabbits with the three rabbit groups in this report.

	Literature (17)	Control (group 1)	With Ezetemibe (group 2)	Without Ezetemibe (group 3)
Weight (kg)	2.59	3.1 ± 0.26	3.11 ± 0.16	3.05 ± 0.2

E/A *	2.19	1.80 ± 0.51	1.92 ± 0.37	1.55 ± 0.49

E'/A'‡	NA	1.93 ± 0.42	1.82 ± 0.22	1.26 ± 0.62

S'‡	NA	6.42 ± 2.55	6.55 ± 1.66	4.6 ± 0.69

E/E'	NA	8.91 ± 1.54	8.48 ± 2.15	11.72 ± 5.33

E wave (cm/s)	59	60.6 ± 9.9	59.1 ± 8.5	58.0 ± 9.2

E dec (sec)	NA	87.8 ± 22.5	93.4 ± 18.5	102.1 ± 22.1

IVSd (mm)	2.03	2.85 ± 0.37	2.87 ± 0.35	3.11 ± 0.60

IVSs (mm)	3.05	4.14 ± 0.69	4.25 ± 0.70	3.88 ± 0.33

LVIDd (mm)	14.37	15.14 ± 0.69	14.62 ± 2.38	14.77 ± 2.16

LVIDs (mm)	10.05	10.14 ± 0.37	10.5 ± 2.07	10.77 ± 1.85

LVFWd (mm)	2.16	3 ± 0.0	2.5 ± 0.75	2.66 ± 0.5

LVFWs (mm)	3.48	4.14 ± 0.89	3.62 ± 0.51	3.77 ± 0.66

FS (%)	30.13	30.85 ± 3.5	28.37 ± 6.36	28.22 ± 4.76

EF (%)	61.29	62.28 ± 5.02	58.37 ± 9.7	58.44 ± 7.76

Ao (mm)	8.26	9.42 ± 0.78	9.25 ± 1.48	9.33 ± 0.86

LA (mm)	9.66	8.85 ± 1.06	8.5 ± 1.41	9.33 ± 2.64

E wave = Peak E velocity, E dec = E wave decelation time IVSd = Intraventricular septum diastole, IVSs = Intraventricular septum systole, LVIDd = Left ventricular internal dimension diastole, LVIDs = Left ventricular internal dimension systole, LVFWd = Left ventricular free wall diastole, LVFWs = Left ventricular free wall systole, FS = Fractional shortening, EF = Ejection fraction, Ao = Aortic root diameter, LA = Left atrial diameter.

From: Fontes-Sousa, et al. M-mode and Doppler echocardiographic reference values for male New Zealand white rabbits. *AJVR *2006;67:1725-1729 (17).

* =p < 0.05 for group 1 vs. group 3

‡ =p < 0.05 for group 1 and group 2 vs. group 3

**Figure 1:**
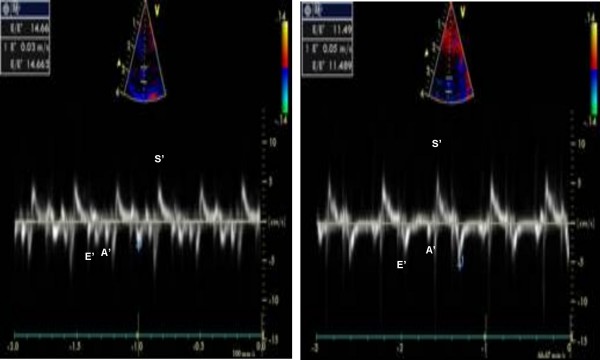
**Tissue Doppler images from the septal wall of the ventricle**. Tissue Doppler images (bottom) obtained from the Septal wall (top) in two different rabbits on hypercholesterolemic diet with (right) and without ezetimibe (left). E' = peak early diastolic velocity, A' = peak atrial velocity, S' = peak systolic velocity.

**Figure 2:**
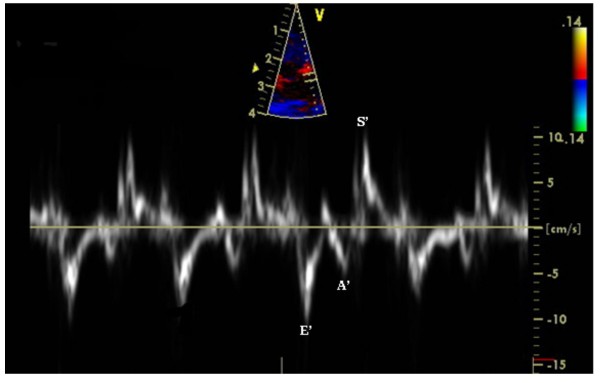
**Tissue Doppler image from the lateral wall of the ventricle**. Tissue Doppler image (bottom) obtained from the lateral wall of the ventricle (top) in a control rabbit. E' = peak early diastolic velocity, A' = peak atrial velocity, S' = peak systolic velocity.

**Figure 3:**
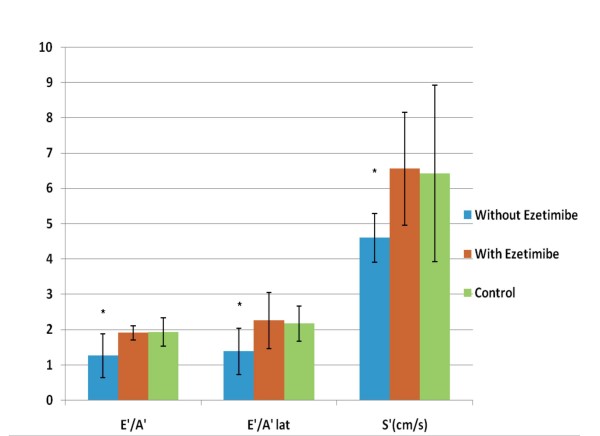
**Myocardial Function by Subgroups**. E'/A' (septal wall), E'/A' lat (lateral wall) and S' measurements in hypercholesterolemic rabbits with and without ezetimibe and control rabbits. *p < 0.05 for group without ezetimibe vs. with ezetimibe and control.

Several TDI derived parameters were significantly different among the groups (Figures 1, 2). The E'/A' ratio measured in the septal wall was higher in group 1 (1.93 ± 0.42) and group 2 (1.82 ± 0.22) compared to group 3 (1.26 ± 0.62) (p = 0.03). Similar values and statistical differences were noted for the lateral wall for group 1 (2.16 ± 0.5) and group 2 (2.25 ± 0.8) compared to group 3 (1.38 ± 0.66; p = 0.02) (Figure 3). However, the mitral Doppler inflow velocity showed no difference with regards to E wave acceleration or deceleration. Also, there was a borderline significant decrease in the E/A ratio in group 3 when compared to groups 2 and 1 respectively (1.55 ± 0.49 vs. 1.92 ± 0.37 and 1.80 ± 0.51; p = 0.07 and p > 0.1; student t-test).

Group 1 and group 2 had higher S' measurements compared to group 3 respectively (6.42 ± 2.5 and 6.55 ± 1.66 vs. 4.6 ± 0.69; p = 0.03). Also, Group 3 was noted to have an elevated E/E' ratio (11.5 ± 4.9) in comparison to both group 2 (8.72 ± 2.0) and group 1 (8.91 ± 1.54) with a trend towards statistical significance (respectively p = 0.07; p = 0.08; student t-test).

### Serum and Tissue Cholesterol

Peak serum cholesterol levels were 495 ± 305 mg/dl vs. 114 ± 95 mg/dl and 87 ± 37 mg/dl; p < 0.01. Myocardial cholesterol content was also significantly greater in group 3 vs. group 2 (0.105 ± 0.04 mg/g vs. 0.068 ± 0.02; p = 0.05; one tailed student t-test).

### Correlation Between Echocardiography and Cholesterol Levels

There was a significant negative correlation between serum cholesterol levels and the TDI derived measurements including E' (r^2 ^= 0.28, p = 0.009), S' (r^2 ^= 0.16, p = 0.04), E'/A' (r^2 ^= 0.37, p = 0.001) with a positive correlation with E/E' (r^2 ^= 0.24, p = -0.01). Also, there was a significant negative correlation between myocardial cholesterol content and E' (r^2 ^= 0.16 p = 0.04) and S' (r^2 ^= 0.15, p = 0.05) with a positive correlation with E/E' (r^2 ^= 0.41, p < 0.01). However, there was no correlation between serum or myocardial cholesterol levels and the E wave peak velocity, the peak A wave velocity, E/A ratio or ejection fraction (Table 2).

**Table 2:** Correlations (r^2^) between TDI, 2D and Doppler echocardiographic parameters with serum and myocardial cholesterol

	TDI parameters				2D and Doppler parameters		
	**E'/A'**	**E'**	**S'**	**E/E'**	**E wave (cm/s)**	**E/A**	**EF (%)**

Serum Cholesterol	0.37*	0.28*	0.17*	0.24*	0.01	0.004	0.004

Myocardial Cholesterol	0.008	0.16*	0.15*	0.41*	0.02	-0.01	0.004

*=p < 0.05

## Discussion

This study showed a statistically significant *in vivo *correlation between myocardial cholesterol loading and myocardial dysfunction as measured via advanced echocardiographic imaging but not via traditional echocardiographic parameters. Prior studies on lipid lowering in the myocardium have primarily evaluated the effects of triglycerides, but only a few have considered the effects of cholesterol on myocardial function [19]. The toxic effects of cholesterol on the myocardium have been investigated by various methods and have been described as "lipotoxic cardiomyopathy" [20], "cholesterol cardiomyopathy" [19] and "cardiac steatosis" [21]. The data presented suggest that cholesterol accumulates in the myocardium and affects both systolic and diastolic function; this was inhibited by reducing cholesterol absorption.

Zaca *et al *evaluated the role of cholesterol accumulation in the myocardium of a dog model by using hemodynamic measurements [22]. Also, Huang *et al*, Chiu *et al *and Wu *et al *have all performed *ex-vivo *physiologic studies of myocardial lipid content by histology and molecular analysis [19,20,23]. Huang used a very similar model of hypercholesterolemia in rabbits and demonstrated systolic and diastolic dysfunction with individual myocytes in a perfusion chamber and associated this finding with reduced levels of sarcoplasmic/endoplasmic reticulum Ca2_-ATPase (SERCA)-2 mRNA; we confirmed their bench-top findings with TDI. Chiu and Wu on the other hand used genetically modified mice models to obtain very high cholesterol levels and they also found myocardial dysfunction in their respective ex-vivo models. In our study, we were able to confirm similar findings of myocardial dysfunction via a non-invasive method in live subjects.

Human studies demonstrating cardiac lipid accumulation have been technically limited [24]. A recent human study found myocardial triglyceride accumulation and impaired diastolic function via magnetic resonance spectroscopy [21]. However, in that study data was not obtainable in over 25% of the subjects because of technical limitations. In our study, as well as human studies, the image quality with echocardiography is consistent and readily obtainable. Moreover, most studies [21,25] evaluated triglyceride, rather than cholesterol levels and focused on the potential alterations in myocardial metabolism.

The use of statins for congestive heart failure has yielded conflicting results [25] with some studies demonstrating improved LV function [26], symptomatology, clinical outcomes [27] and mortality [28]. However, most studies have tackled this problem at advanced stages of heart failure, but we demonstrated that measurable differences can occur at the very earlier stages of LV dysfunction.

In our study, we lowered cholesterol with ezetimibe since it has no known pleiotropic effects other than decreasing cholesterol absorption [29]. This decrease was associated with similar cholesterol levels as the chow fed group and hence similar myocardial function measurements. Thus, we were able to establish correlations between serum and myocardial cholesterol levels and TDI markers of myocardial function. This supports our hypothesis of a potential role of cholesterol infiltration of the myocardium in the pathogenesis of cardiac dysfunction. Thus, TDI measurements may be used as a marker of cardiac cholesterol infiltration in metabolic diseases such as diabetes, hypercholesterolemia and metabolic syndrome.

### Potential Mechanisms

The mechanism by which hypercholesterolemia alters myocardial function is not clear, and it may overlap with its established effects on the coronary macro and microvasculature (A). Other studies have demonstrated the deleterious effect of high glucose levels on myocardial function with microvascular dysfunction and progressive LV remodeling being implicated for such changes [30]. It has been suggested that the deposition of cholesterol in the myocardium may cause a cholesterol myopathy similar to other infiltrating diseases [31]. Alternatively, myocardial metabolism may shift ATP production from a glucose based to a free fatty acid state [32] and flood the Krebs cycle to create more free radicals that injure the myocardium [33,34].

### Limitations

This study does not establish causality between higher cholesterol and myocardial function. We used an aggressive dietary regimen to attempt to produce effects that may otherwise take years to develop in humans. Extrapolating these findings to the clinical arena may be relevant based on a lone study that found similarities between cholesterol accumulation in lipotoxic rat hearts to human transplanted hearts [35].

## Conclusion

A high cholesterol diet is associated with decrease in both systolic and diastolic cardiac function in rabbits as measured by TDI, while ezetimibe is protective by its capacity to decrease cholesterol absorption. The mechanism by which cholesterol alters the myocardial function remains to be established by further research possibly with imaging and biochemical studies of the myocardium.

## Competing interests

The authors declare that they have no competing interests.

## Authors' contributions

JR conceived and designed the study, analyzed and interpreted the data and drafted the manuscript. AP played a key role in drafting, reviewing and revision of the manuscript. AV participated in data analysis and interpretation. PK participated in collection, analysis and interpretation of data and drafting of the manuscript. GSA played a key role in drafting and revision of the manuscript and also gave the final approval of the version to be published.
